# Circulating Exosome Cargoes Contain Functionally Diverse Cancer Biomarkers: From Biogenesis and Function to Purification and Potential Translational Utility

**DOI:** 10.3390/cancers14143350

**Published:** 2022-07-10

**Authors:** Megan I. Mitchell, Junfeng Ma, Claire L. Carter, Olivier Loudig

**Affiliations:** 1 Center for Discovery and Innovation, Hackensack Meridian Health, Nutley, NJ 07110, USA; megan.mitchell@hmh-cdi.org (M.I.M.); claire.carter@hmh-cdi.org (C.L.C.); 2 Department of Oncology, Lombardi Comprehensive Cancer Center, Georgetown University Medical Center, Washington, DC 20007, USA; jungfeng.ma@georgetown.edu

**Keywords:** extracellular vesicles, exosomes, circulating biomarkers, transcriptomics, proteomics, lipidomics, early cancer detection

## Abstract

**Simple Summary:**

The indolent nature of some cancers makes early detection challenging, as such significant effort is placed on identifying circulating cancer biomarkers using minimally invasive, highly sensitive diagnostic assays. Biological fluids contain small extracellular vesicles including exosomes, which have many tissue origins. Cancer cells increase production and release of exosomes in the circulation to deliver biologically active compounds that can reprogram recipient cells, which potentially represent a valuable source of biomarkers. In this review, we evaluate the biogenesis of exosomes to understand how cancer cells alter their production and how their molecular cargoes interact with the microenvironment. Next, we provide a comprehensive inventory of techniques available for exosome purification, as this represents the most critical aspect for sensitive and specific evaluation of their biomarker content. Finally, we provide a current evaluation of their use as human cancer biomarkers.

**Abstract:**

Although diagnostic and therapeutic treatments of cancer have tremendously improved over the past two decades, the indolent nature of its symptoms has made early detection challenging. Thus, inter-disciplinary (genomic, transcriptomic, proteomic, and lipidomic) research efforts have been focused on the non-invasive identification of unique “silver bullet” cancer biomarkers for the design of ultra-sensitive molecular diagnostic assays. Circulating tumor biomarkers, such as CTCs and ctDNAs, which are released by tumors in the circulation, have already demonstrated their clinical utility for the non-invasive detection of certain solid tumors. Considering that exosomes are actively produced by all cells, including tumor cells, and can be found in the circulation, they have been extensively assessed for their potential as a source of circulating cell-specific biomarkers. Exosomes are particularly appealing because they represent a stable and encapsulated reservoir of active biological compounds that may be useful for the non-invasive detection of cancer. T biogenesis of these extracellular vesicles is profoundly altered during carcinogenesis, but because they harbor unique or uniquely combined surface proteins, cancer biomarker studies have been focused on their purification from biofluids, for the analysis of their RNA, DNA, protein, and lipid cargoes. In this review, we evaluate the biogenesis of normal and cancer exosomes, provide extensive information on the state of the art, the current purification methods, and the technologies employed for genomic, transcriptomic, proteomic, and lipidomic evaluation of their cargoes. Our thorough examination of the literature highlights the current limitations and promising future of exosomes as a liquid biopsy for the identification of circulating tumor biomarkers.

## 1. Introduction

Despite recent advances in diagnostic and therapeutic strategies, cancer continues to significantly decrease life expectancy, and globally remains among the top five leading causes of death [1]. The World Health Organization (WHO) and the Centers for Disease Control and Prevention (CDC) estimate that cancer is amongst the top two leading causes of death in developed countries, including the United States [2,3].

Advancements in the early detection of cancer have demonstrated the benefits of routine screening, as evidenced by decreased mortality rates, and have also led the way for the design of novel screening technologies and prognostic assays [4]. Early detection, to date, remains the most efficient way to improve cancer outcomes because advanced disease is inextricably correlated with tumor cell dissemination, treatment failure, and high recurrence rates, which are associated with ~90% of cancer deaths [5,6,7]. Unfortunately, many somatic cancer types (e.g., lung cancer, pancreatic cancer, brain cancer, ovarian cancer, and lymphoma), are still very difficult to detect early, often due to the indolence of their symptoms and the late onset of disease, and are generally associated with poor outcomes [8]. Thus, clinical efforts have been aimed at initiating early screening of patients who are at risk for these cancers (due to family history, risk exposure, etc.), while research efforts have been aimed at identifying circulating molecular biomarkers with the intent to develop non-invasive and universal early detection assays (i.e., liquid biopsies) [9,10]. Of relevance, single screening biomarkers such as prostate-specific antigen (PSA) for prostate cancer, the cancer antigen 125 (CA125) for ovarian cancer, the cancer antigen 15-3 (CA15-3), the carbohydrate antigen 19-9 (CA19-9), and the carcinoembryonic antigen (CEA) for breast cancer are routinely used in clinical cancer diagnostics [11,12,13,14]. Although single protein screening biomarkers are practical, generally blood-based, and minimally invasive, they display strong limitations due to high rates of both false-positive and false-negative results, confounding the accurate detection of disease and highlighting the need for exploration of other circulating biological materials as early prognostic tools [15,16,17,18,19,20].

In the last two decades, cancer genetics has improved our understanding of the hallmarks of cancer, along with the alterations associated with cancer development and progression [21,22,23]. The discovery of mutational drivers of carcinogenesis specific to each cancer type (i.e., BRCA-1/-2 for breast cancer, EGFR or KRAS for lung cancer, TP53 and RB1 for pancreatic cancer) has provided novel targets for blood-based fine-tuned molecular detection of specific tumors (breast cancer, prostate cancer, colorectal cancer, thyroid cancer, and lung cancer) [24,25,26,27,28]. The term “liquid biopsy” has been exemplified by the development of state-of-the-art methodologies for detecting circulating tumor cells (CTCs) [29,30,31] and circulating tumor DNA (ctDNA) [32,33,34,35]. For the detection of CTCs, which are released into the circulation by primary tumors during early stages of tumor growth and vascularization [36,37,38], novel technologies have been developed to detect as low as one CTC for every 1 × 10^9^ red blood cells per milliliter of peripheral blood [39], by targeting their physical properties (i.e., size and/or density), using size-based selection/discrimination methods [40,41] and/or immunoaffinity-based selection methods that target their distinct surface tumor markers (e.g., antibodies targeting general CTC markers such as EpCam or more specific markers such as prostate-specific membrane antigen (PSMA) for prostate specific CTCs) [42,43,44,45]. Although CTC panels (e.g., CellSearch^®^ CTC test) have been approved for clinical use for the detection of metastatic breast, prostate, and colorectal cancers, a significant lack of standardization between the different commercial technologies, including immunomagnetic and microfluidic variations, has hampered their widespread application [46,47,48].

In contrast, and advantageously, the screening for circulating tumor DNA (ctDNA), which relies on quantitative PCR, is highly specific and sensitive, and is based on the detection of unique somatic mutations harbored by the genomic DNA of dying circulating tumor cells and dying tumor cells (cell death, immune response, etc.) [49,50,51]. Screening panels for ctDNA have already been commercialized (e.g., Signatera tumor-informed assay [52]) for molecular monitoring of residual disease, response to therapeutic treatment, and recurrence. Considering the value of ctDNA for the detection of cancer, high-throughput targeted next-generation sequencing panels have been developed for clinical screening of ctDNA from circulating genomic DNA (i.e., Illumina TruSight cancer panel) [53,54]. However, sequencing depth remains an important limiting factor in detecting circulating genomic mutations at ultra-low levels of representation [55]. Although extremely accurate and sensitive, the methods developed for purification and detection of CTCs and ctDNA are limited by the amount of circulating material [56,57]. In fact, many studies have shown that both CTCs and ctDNA are often undetectable in a percentage of cancers [58,59,60], and both require knowledge of the mutations (e.g., protein or DNA) for tumor screening [61,62].

In contrast to CTCs and ctDNA, which are in very low abundance at early stages of tumor development, tumor secreted nanoparticles, also known as extracellular vesicles (EVs) or exosomes, have been shown to represent a robust, continuously produced, and unique source of biomarkers actively secreted by their tumor cells of origin [63]. Advantageously, membrane-bound stably encapsulated biomarkers (i.e., intact RNAs, fragmented gDNA, and intact surface and cytosolic proteins) are packaged and released in a higher abundance by tumor cells into both the microenvironment and the surrounding biofluids [64,65,66,67], and particularly into the circulation [68]. Although the nucleic acid and protein/lipid ratios of exosomes do not directly recapitulate those of their cell of origin, their biogenesis and content is representative of transformed cells, containing unique transcriptomic alterations and proteomic and lipidomic functional characteristics. Thus, exosomes and other EVs show great promise as molecular reservoirs for liquid biopsy in both cancer diagnosis and monitoring, but also as specifically targeted beacons for the delivery of clinical therapeutics [69,70,71].

This review is focused on understanding why exosomes are a unique and purifiable source of circulating tumor biomarkers. The detailed description of their biogenesis and their content will provide clues on why and how they hold both an intriguing and promising future as a minimally invasive source of liquid biopsy, as their presence in all tested biofluids, including blood, exhaled breath condensate (EBC), urine, sweat, saliva, and cerebral spinal fluid (CSF), provides localized and targetable biological material for clinical screening. Importantly, we will evaluate the technological landscape for their purification, including both global and targeted purification approaches. We will discuss the genomic, transcriptomic, and proteomic biomarker aspects of their cargo, which offer a unique and remote snapshot of tumor cell by/bio-products. By evaluating the ongoing technological efforts and weighing the current limitations, we provide a realistic view of the application of exosome biomarkers for the detection and monitoring of human cancers (e.g., early detection, treatment response, recurrence, and metastasis). 

## 2. Extracellular Vesicles (EVs): A Journey from Discovery to Clinical Utility

The first description of EVs can be traced back to 1946, to the studies of Erwin Chargaff and Randolph West on blood coagulation [72,73]. They were the first to describe a platelet-free coagulation component from plasma which could be sedimented into a pellet by high-speed centrifugation (31,000× *g*) and could inhibit blood clotting [74].

The definition of exosomes was introduced in complementary seminal papers from Philip Stahl and Rose Johnstone’s laboratories in 1983 [75,76]. Stahl published stunning electron microscopic images which demonstrated the mechanical process of exosome release from the lumen of MVBs upon fusion with the plasma membrane, which helped define this novel intracellular sorting pathway then referred to as the exosome secretion pathway [75]. Functionally, Rose Johnstone and co-workers suggested that exosomes served as a cellular waste disposal mechanism, wherein they showed that under different cellular stressors the presence of the transferrin receptor on exosomes was altered at different times, thus suggesting that exosomes provided a major route for the shedding of obsolete membrane proteins [77]. However, many contradicting papers were published demonstrating the lateral cell–cell diffusion/transfer of proteins and lipids within the membranes of exosomes (i.e., membrane fluidity) [78], along with the identification of functional enzymes [79] and other active components (i.e., the tetraspanins, Rab4, and ARF [80]), suggestive of their active biological function. Additionally, several papers demonstrated that the production of exosomes and their composition could be altered at different stages of disease, being increased during transient brain ischemia, myocardial infarctions, angina, and Crohn’s disease, wherein exosomes were shown to act as functional activators [81,82,83].

**Figure 1:**
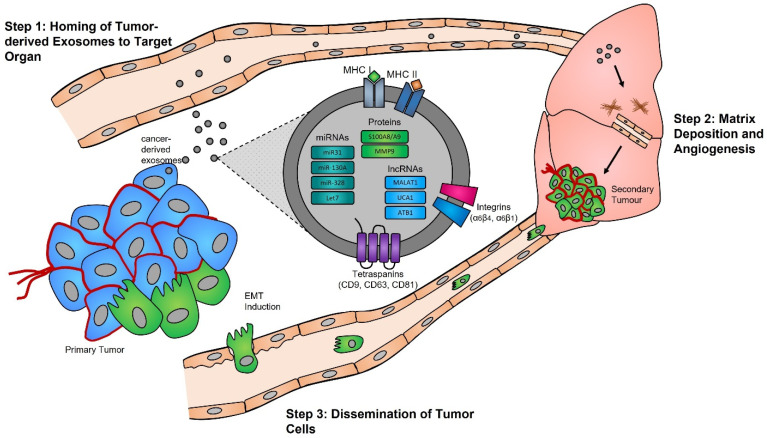
Exosomes and pre-metastatic niche formation. Exosomes released from primary tumors into the circulatory system specifically home to distant target organs (step 1). Upon their arrival, tumor-derived exosomes actively prepare the pre-metastatic niche through myofibroblast activation, induction of angiogenesis, and ECM remodeling (step 2). Local invasion of the primary tumor by cancer cells is followed by their intravasation into the tumor vasculature. These cancer cells survive and travel within the circulatory system, and upon their arrest in capillaries at distant sites, they extravasate into the parenchyma of target organs to commence metastatic colonization (step 3).

Functional studies, many of which were conducted in the early 2000s, have highlighted the importance of the exosome cargoes and their functions as unique biological devices. Exosome proteomics, lipidomics, and transcriptomics analyses have led to the discovery of unique exosome cell-specific cargoes [84,85,86,87], confirming that they transport active proteins and also, importantly, carry unique and intact RNA molecules (i.e., mRNAs, miRNAs, and lncRNAs) that can post-transcriptionally modify the fate and behavior of recipient cells [88,89,90]. The study by Skog et al. demonstrated that the RNA found in glioblastoma-derived exosomes contains a “snapshot” of the cellular transcriptome of their cells of origin at a specific point in time [91]. With an ever-growing number of studies demonstrating the powerful and oncogenic effects of tumor exosomes, important breakthroughs such as the mechanistic description of metastatic dissemination have highlighted the unique intercellular communication properties of exosomes [92,93,94,95]. Jang et al. demonstrated in their study that the enhanced release of breast tumor-derived exosomes is critical for the education of pre-metastatic niche cells, which accommodate the colonization of circulating cancer cells from primary tumors [96,97]. This has in part been explained by the “seed and soil” hypothesis proposed by Stephen Paget [98], wherein exosomes carry oncogenic signals (i.e., mRNA, miRNA, and protein) specifically packaged by primary tumors’ (i.e., the “seed”) to prime target sites (i.e., the “soil”) prior to the dissemination of tumor cells, a process by which secondary tumors can prosper in a suitable microenvironment [99,100] (Figure 1).

An important potential application of circulating tumor exosomes that has been extensively studied is their targeted purification for analysis of their content and identification of tumor biomarkers, including exosome studies of breast cancer [101], lung cancer [102], colon cancer [103,104], and pancreatic cancer [105,106]. Although many options are available for the purification of exosomes from biofluids, very few methods have been proven to provide both the sensitivity and specificity required for detecting low abundance tumor-specific circulating exosomes. Technological advancements aimed at enhancing tissue/cell-specific purification of exosomes from biofluids has significant potential to provide ultra-sensitive molecular assays for detection and monitoring of all human diseases, in particular cancer.

## 3. Current Exosome Isolation Techniques—Advantages and Limitations

The technologies and tools that have been used to purify exosomes from blood and other biofluids have evolved greatly in the past two decades, and this evolution has been driven by the need to accurately assess their biological function, but also and most importantly to decipher their molecular content, with a particular focus on tumor exosome biomarkers [107]. Isolation technologies are still evolving and include: (1) ultracentrifugation, (2) filtration, (3) precipitation, (4) immunoaffinity, and (5) microfluidics that often combine size separation and immunoaffinity [108]. Although some of these approaches are considered state-of-the-art for the isolation of exosomes, many of them have inherent limitations not adapted to the nanomolecular specificity and sensitivity required for purification of cell-type specific exosomes [109].

### 3.1. Ultracentrifugation-Based Isolation

Exosome isolation by means of ultracentrifugation is still the most widely used approach and is considered the gold standard for sedimentation of exosomes [110,111]. To date, it is estimated that ultracentrifugation accounts for ~56% of all exosome isolation techniques, as it is demonstrated to provide high-purity exosome fractions from biofluids [112]. Currently, two protocols for ultracentrifugation-based exosome isolations are used, including either differential ultracentrifugation or density gradient ultracentrifugation [113].

Differential ultracentrifugation: consists of a series of sequential centrifugation cycles at different centrifugal forces and durations [113]. The initial preparation of biofluids typically starts with several short, low-speed centrifugation steps (i.e., 2000× *g* for 10 min followed by 10,000 × *g* for 30 min [110]) that are necessary for the removal of contaminating cellular debris and larger microvesicles [114]. Next, a first round of ultracentrifugation is carried out at ~100,000× *g* for 90 min to generate an exosome pellet, which is washed with an appropriate isotonic buffer such as phosphate buffered saline (PBS) to remove protein and other soluble molecules. Subsequently, a second ultracentrifugation round (i.e., at ~100,000× *g* for 90 min) is performed to obtain the final exosome pellet, which is generally re-suspended in PBS and stored at −80 °C to await downstream analyses. Studies have also shown that exosomes purified by ultracentrifugation can be stably stored at 4 °C, where they maintain their intactness and retain their function for up to 20 months [115].

Density-gradient ultracentrifugation: (standard or isopycnic) has recently gained popularity because studies have shown that it increases the purity of exosome preparations [116]. Here, the separation of exosomes is achieved by the layering of a liquid sample as a narrow band on top of a medium, typically sucrose or iodixanol [117]. With the application of centrifugal force (i.e., at ~100,000× *g* for up to 18 h), the gradient allows for the separation of solutes, including exosomes, and their specific sedimentation into several distinct solute layers. After centrifugation, individual 1 mL gradient fractions are manually collected using a pipette [118]. The separated exosome fraction is then diluted with 1x PBS and subjected to a second round of ultracentrifugation (i.e., at ~100,000× *g* for ~70 min [116]). Similar to standard ultracentrifugation, the resulting exosome pellet is resuspended in PBS and stored at −80 °C. The biggest limitation when selecting density-gradient ultracentrifugation over differential ultracentrifugation is that the sample volume capacity for exosome isolation is greatly reduced in the former (~5% of the centrifuge tube capacity) [119].

Although ultracentrifugation remains the gold-standard for sedimentation of exosomes devoid of other EVs (i.e., larger sized microvesicles, cell debris, proteins) and lipoprotein contaminants, it requires expensive instrumentation, but most importantly only provides a bulk exosome isolate from a specific biofluid, rather that separating cell/tissue-specific exosome sub-populations from a biofluid [120]. Additionally, recent studies have reported that repeated rounds of ultracentrifugation reduce exosome yield and that extended and repeated high shear forces from high-speed ultracentrifugation adversely affect their integrity [121,122].

### 3.2. Size-Based Separation and Isolation

To date there are three main methods for the size-based isolation of exosomes, which include ultrafiltration, sequential filtration, and size-exclusion chromatography (SEC). These methods have been developed to bypass extended ultracentrifugation times and to facilitate the fractionation of exosomes from large biofluid volumes.

Ultrafiltration: relies on the use of 10–100 kDa molecular weight cutoff (MWCO) filters, which are most often utilized to reduce large sample volumes (i.e., urine or cell culture supernatants) by concentration to smaller more manageable volumes that often simplify downstream applications [123]. Currently, several commercially available kits are on the market which have been proven to provide pure populations of exosomes from biofluids. These include the Qiagen exoEasy kit, which allows isolation of exosomes from volumes up to 4 mL of plasma or 16 mL of cell culture media [124]; the Amicon^®^ Ultra-15 centrifugal filter tubes (MilliporeSigma, Burlington, MA, USA), which allow filtration of up to 15 mL [125]; and the ExoLution^®^ plus platform from Exosome Diagnostics, which allows the processing of 3 mL of plasma [126].

Sequential filtration:, unlike standard ultrafiltration, typically includes three steps [127]. First, larger cells and cellular debris are filtered out using standard filtration methods (0.2 µm filters). Second, free proteins are depleted by tangential flow filtration, where biofluids are passed parallel to the filter, allowing for continuous filtration and reduced membrane clogging [128]. Third, exosomes are sorted out with the use of a track-etched filter membrane (e.g., Whatman/GE Nucleopore 50, 80, 100, or 200 nm diameter membranes), allowing for size-based fractionation of exosomes [129].

Size exclusion chromatography (SEC): relies on passing a biofluid (known as the mobile phase) through a porous gel filtration polymer (i.e., the stationary phase [130]). The stationary phase of the chromatography column can be packed with different types of polymers (e.g., agarose, polyacrylamide, crosslinked dextrans, or allyldextran), the nature of which allows for differential elution of the sample into size-based fractions (i.e., larger particles travel faster and elute first, followed by smaller particles and finally non-membrane bound proteins [131]). In terms of its use for exosome isolation, SEC, when performed at lower flow rates, has been shown to possess several advantages, including reproducibility, cost efficiency, and the isolation of undamaged exosomes [121]. SEC significantly outcompetes other filtration-based isolation techniques in that it effectively removes protein contaminants, thereby yielding highly pure exosome fractions [132]. The isolation, purification, and enrichment of exosomes using SEC has been successfully used with a variety of biological fluids, including serum and plasma [133], cerebrospinal fluid [134], bovine and human milk [135], saliva [136], urine [137], synovial fluid [138], tears [136], nasal lavage [139], and seminal fluid [140].

Although great purity is achieved via SEC, which can accommodate large volumes of sample (i.e., up to 100 mL using the qEV100 column from IZON), this approach does not allow for separation of different exosome subpopulations or for separation of exosomes from other vesicles of the same size; thus, contamination by lipoproteins and other microvesicles cannot be ruled out [141].

### 3.3. Polyethylene Glycol (PEG) Precipitation-Based Isolation

The isolation of exosomes can also be achieved by precipitation by the addition of an aqueous polyethylene glycol (PEG) solution to a biofluid. Here, PEG coats the surface of exosomes and other microvesicles, facilitating the formation of exosome–PEG aggregates [142]. Exosomes are trapped in this porous microstructure, and are then precipitated (e.g., ExoQuick^®^) by low-speed centrifugation at 1500× *g* [143]. This method results in the isolation of exosomes within a size range consistent with ultracentrifugation; however, as PEG is a non-specific “coagulant”, it also results in the co-precipitation of soluble non-exosomal proteins, immunoglobulins, and lipoproteins, which significantly limits the purity of the final exosome pellet [144] as they carry unwanted biological material (i.e., proteins and nucleic acid species) [145]. The advantages of this procedure are that it is inexpensive, requires little to no training, and it allows for high-throughput processing of samples with little damage to exosomes. Although PEG-based precipitation results in high yield, low purity exosomes, studies have shown that when it is sequentially combined with an immuno-purification (such as anti-CD63 assay), the exosome fraction purity may be enhanced [146], making it an attractive approach for initial, fast, and crude isolation of exosomes [147].

### 3.4. Immunoaffinity-Based Isolation

In recent years, immunoaffinity-based capture has become one of the preferred methods for the isolation of exosomes from biofluids [148]. This technology allows for direct separation of exosomes via immobilized antibody targeting of membrane-surface specific exosomal proteins [149,150,151]. Most, if not all, commercially available immunoaffinity-based isolation kits are tailored with antibodies (either alone or in combination, i.e., a pan-exosome panel) targeting common exosome surface markers, such as the tetraspanins (CD9, CD63, and CD81) [152]. Other assays also include antibodies targeting markers such as epithelial cellular adhesion molecule (EPCAM) [153] and exosome-binding molecules such as heat shock proteins (HSP70, HSP90) [154] or heparin [155]. A large number of biotechnology companies, including MBL (EXOCAP, Sunnyvale, CA, USA), Systems Bioscience (ExoFlow, Palo Alto, Santa Clara, CA, USA), FujiFilm (MagCapture™, Wako Chemicals, Richmond, VA, USA), and BioLegend (MojoSort™, San Diego, CA, USA), provide ready-to-use immunoaffinity assays for exosome isolation [145] that utilize antibody-coated magnetic beads for the capture of exosomes, as they are convenient, allow rapid and easy magnetic isolation of exosomes, and ultimately allow for their concentration into a small volume [156]. Although the selection of exosomes from a biofluid, with antibodies targeting exosome surface proteins, provides the state-of-the-art selection, very few provide assays that allow for the gentle release of captured exosomes [149]. Another critical issue with magnetic beads, as we determined by quantitative PCR of small-RNA contaminants, is that they can bind small-RNAs and EVs (containing small-RNAs), which may be detrimental for downstream analyses, especially when analyzing the small-RNA content of exosome sub-populations that are in low abundance [149]. We hypothesized that the polymer coating is positively charged and the pore sizes between adjacent streptavidin/carboxyl molecules may be large enough to allow for non-specific binding of small biological material (i.e., nucleic acids, proteins, and lipids), creating a non-specific signal background that will interfere with subsequent analyses and the identification of exosome tumor biomarkers (i.e., transcriptomic signatures). To circumvent these limitations, we recently developed an assay that we termed EV-CATCHER (Extracellular Vesicles Capture by AnTibody of CHoice and Enzymatic Release), which greatly reduces non-specific binding of small-RNAs and EVs, which can be customized with any antibody, and which allows for the mild enzymatic release of intact EVs [149].

Overall, immunoaffinity-based capture generally reduces exosome yield (as only antibody-recognized exosomes are captured) and is generally more expensive; however, when coupled with an initial step of ultracentrifugation or ultrafiltration to concentrate exosomes, it can result in higher exosome purity and may help separate exosomes of specific cellular origin from the bulk exosome preparation. Many studies on lung [157], breast [158], colorectal [159], liver [160], and many other cancers have already demonstrated that with higher exosome purity and selectivity, the analysis of their molecular cargo can provide more specific and reproducibly detectable biomarkers [161,162]. Thus, the immuno-affinity separation of unique tumor exosome cargoes from biofluids has potential to lead to novel cancer diagnostic assays [163].

### 3.5. Microfluidics-Based Isolation

Microfluidics-based exosome isolation systems have become a sought-after nanotechnology for separating exosomes from other nanosized bioparticles. This technology provides high-speed, high-throughput, ultra-precise, and cost-efficient isolation of exosomes [164]. Emerging chip-based microfluidic exosome isolation techniques, including standard research laboratory-based approaches developed by Duke University using acoustic microfluidics (i.e., acoustofluidics) for the separation of exosomes from whole blood [165], the exosome total isolation chip (ExoTIC) device developed by Liu et al., and the ExoChip developed by Kanwar et al., have already been marketed for efficient isolation of high-yield, high-purity intact exosomes [166,167]. The ExoTIC device utilizes a simple filtration-based approach wherein EV-containing biofluids, such as culture media, plasma, and urine, are passed through a nanoporous membrane [166]. During this process, free-floating proteins, nucleic acids, and lipids are washed out, and the enriched exosomes (30–200 nm in size) can be collected from the membrane using a standard pipette [166]. In comparison to ultracentrifugation and precipitation-based exosome isolation, the ExoTIC device has been shown to possess undisputed advantages, which include the constant flow of clinical specimens through nanochannels that slow the EVs, the separation of large and small vesicles in separate nano-sized chambers via engineered ports, and the presence of antibodies for the capture of exosomes with potential for release [168]. Currently, many of the existing microfluidics-based systems, including acoustic microfluidics, ExoTIC, and ExoChip, incorporate immunoaffinity components for the separation and capture of exosomes by targeting specific surface markers with immobilized antibodies [167,169]. Interestingly, although our recent analyses revealed the strong limitations of magnetic beads for isolating pure exosomes, several microfluidic platforms have incorporated magnetic beads into their systems for the separation of cells from biofluids [166,167]. Currently, some of the most widely used microfluidics-based systems fully integrate size-based separation, immunoaffinity-based separation, and dynamic separation [170]. The most critical issue that microfluidic-based separation technologies have solved is that they avoid the non-continuous separation processes. Indeed, samples are processed from start-to-finish in a single run, in a closed loop, with limited user-interference or introduction of contaminants, which helps maintain yields that are otherwise reduced by the repeated washes performed during sequential ultracentrifugation [121]. Several problems still remain, however, which are similar to those mentioned for immunoaffinity-based isolation techniques and include the need for high immunoaffinity and the requirement for highly specific and sensitive antibodies [171]. Additionally, while microfluidic systems require less hands-on manipulation, they require expensive equipment and complex nano-sized chips that are often difficult to mass-produce [172]. Despite these challenges that remain to be solved, microfluidics-based purifications offer a promising technology for all-in-one chip-based robust isolation and characterization of circulating tumor exosome biomarkers.

### 3.6. Exosome Sorting by Fluorescence Activated Cell Sorting (FACS)

The separation of cells by means of flow cytometry is commonplace [173,174,175]. However, because exosomes are 30–150 nm in size, they fall under the threshold of even the most sensitive “reference noise” region of flow cytometers [176,177]. Nevertheless, selective sorting of antibody-labeled exosome sub-populations by FACS shows great potential for cancer biomarker screening and discovery. As such, extensive research efforts have been applied to the development of high-fidelity-based FACS systems for sorting exosomes [178,179,180] for subsequent proteomic and transcriptomic analyses [181]. One of the biggest advancements has been the development and marketing of advanced imaging flow cytometry (iFCM) by ImageStream^x^ (ISx, EMD Millipore, Seattle, WA, USA) [182] which offers significant advantages for the sorting of exosomes and other small EVs when compared to other available technologies [182,183]. Mastoridis et al. demonstrated that by combining iFCM with subset-specific markers, this technology allows for the high-throughput, multiparametric characterization, and functional assessment of exosomes [184]. Another promising avenue for FACS-based sorting and cancer diagnostics based on the presence of unique tumor exosomes in a biofluid (i.e., prostate cancer exosomes) is the multicolor multiplexed in situ proximity ligation, also termed exoPLA, developed in the laboratory of Dr. Kamali-Moghaddam [185,186]. This method relies on four oligonucleotide-conjugated antibodies whose combination uniquely targets a specific type of exosomes (i.e., prostate exosomes) and rolling circle PCR amplification guided by individual pairs of antibodies to generate different fluorescent signals. Ultimately, the combination of three different fluorescent signals allows for the unique detection and sorting of tumor exosomes by flow cytometry [187]. Altogether, while the FACS-based sorting of exosomes is still a relatively new method in comparison to the others described, it provides a promising avenue for highly specific and robust isolation of low-abundance exosome sub-populations and subsequent molecular analyses.

**Figure 2:**
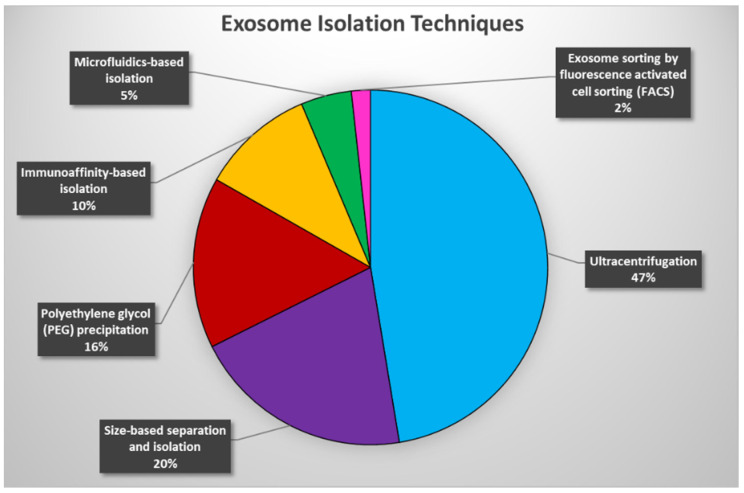
An estimate of the use of exosome isolation techniques over the past 10 years. Pie chart representation of the percentage utilization of each exosome isolation technique extracted from 173 publications spanning the years 2012–2022.

Collectively, while most of the described exosome isolation techniques are currently available and used in different applications (Figure 2), they all possess advantages and disadvantages, which need to be considered prior to initiating exosome analyses. The ideal method for exosome isolation should be relatively simple, efficient, inexpensive, and scalable, but most importantly ultra-sensitive in order to allow for the robust and accurate identification of encapsulated circulating tumor biomarkers and to propel exosome-based liquid biopsies toward ultra-sensitive cancer diagnostics.

## 4. Biogenesis and Function of Exosomes in Normal and Pathological Processes

### 4.1. Biogenesis

**Figure 3:**
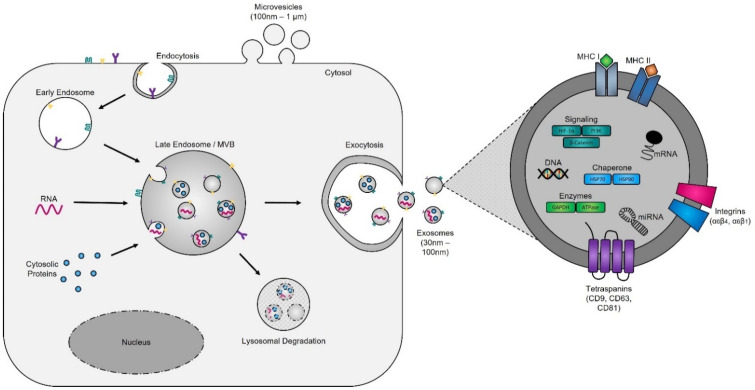
Exosome biogenesis. Exosomes originate from multivesicular bodies (MVBs) (also referred to as late endosomes). The inward budding of the late endosomal membrane around selectively packaged cargo results in the formation of exosomes. The selective packaging of proteins (e.g., tetraspanins, cytoplasmic proteins, and enzymes), nucleic acids (e.g., DNA, RNA, and miRNAs), and lipids (e.g., cholesterol) into exosomes is cell-type dependent and reflects the metabolic status of originating cells. Fusion of MVBs with either lysosomes or the plasma membrane results in either degradation or the release of exosomes into the extracellular matrix, respectively.

Evidence supporting the endosomal origin of exosomes stems from observations that they contain proteins derived exclusively from the cytosol and are devoid of any nuclear proteins [188]. Initial proteomic analyses revealed that while exosomes harbor proteins specific to their cell and tissue of origin, for example integrins (e.g., α6β4, αvβ5, and α6β1 on breast cancer exosomes; αvβ3 and αvβ6 on prostate cancer exosomes; and β4 on pancreatic cancer exosomes), MHC class I and II on immune cell-derived exosomes, as shown for B lymphocytes and dendritic cells [189,190,191], prostate specific antigen (PSA) from prostate cells, asialoglycoprotein receptor 1 (ASGR1) from liver cells [192], microglial proteins (CD11b and CD45) from brain cells [193], placental alkaline phosphatase (PLAP) from placental cells [194,195], and Clara cell protein 16 (CC16) for exosomes produced and released by deep lung cells [196], they also contain proteins common to all exosomes irrespective of their cellular origin, which reflects a highly regulated sorting mechanism [186,197]. For instance, adapter protein ALG-2-interacting protein X (ALIX) and tumor susceptibility gene 101 (TSG101), two proteins extensively characterized for their roles in HIV budding (i.e., release of viral particles from the cell) and MVB formation [198,199], and several tetraspanins (CD63, CD81, CD9, and CD37) are present on all exosomes. The incorporation of specific tetraspanins into exosomes is dependent on cell-type specific needs, as exosomes are involved in a multitude of biological processes, including cell adhesion, motility, invasion, membrane fusion, intracellular signaling, and protein trafficking [200]. To better understand how cancer cells hijack the exosome machinery to regulate the immune response locally, for cell-to-cell communication between neighboring cells, and, as described by Lyden et al. [201], for the successful dissemination of tumor cells, we have divided the endosomal pathway for exosome biogenesis into three major sections (Figure 3).

#### 4.1.1. The Formation of Early Endosomes

Early endosomes were initially defined as the first cellular compartment to receive incoming endocytic vesicle cargoes [202] but are now recognized to be the main sorting station for initiation of the cellular endocytic pathway [203]. The exact mechanisms underlying the initiation of early endosome formation remain unclear; however, it is thought that their membrane is derived from the fusion of primary endocytic vesicles taken up from the extracellular space [204]. The function of early endosomes (i.e., their role in cargo sorting and delivery of vesicles to the plasma membrane) is heterogenous, cell-type dependent, and defined by the proteins present in their cytosol and on their surface [205]. Individually, early endosomes are complex in structure, with their membrane being comprised of a mosaic of protein subdomains, enriched in Rab5, Rab4, Rab11, Arf1/COPI, retromer, and caveolae of cellular origin [206]. Studies of these proteins have demonstrated that they are responsible for the molecular sorting and the direct transport of endosomes once matured to distinct organelles, including the trans-Golgi network and the plasma membrane [207].

#### 4.1.2. The Maturation of Late Endosomes and Formation of Exosomes

**Figure 4:**
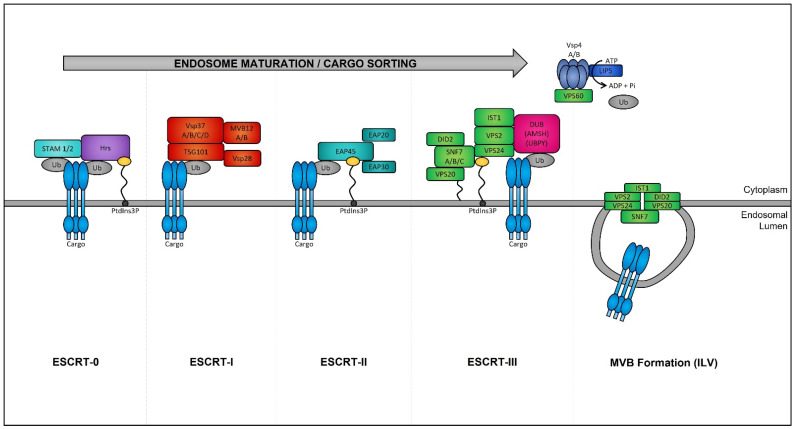
Endosomal sorting complexes required for transport (ESCRT)-dependent MVB formation. ESCRT-dependent MVB formation control the internalization of ubiquitinated proteins into the intraluminal vesicles (ILVs) of MVBs. The ESCRT complex is comprised of a series of sub-complexes which function uniformly during ILV production. ESCRT-0, -I, -II, and -III complexes function consecutively in a stepwise manner to control the selective sorting of ubiquitinated proteins into exosomes.

Late endosomes (also termed MVBs) are described as having a multivesicular morphology because they contain intraluminal vesicles (ILVs) [208]. The membrane of these ILVs contains the majority of V-ATPases (i.e., protein pumps responsible for controlling intracellular and extracellular pH), cholesterol, sphingolipid-rich lipid rafts, clathrin coats, and most importantly the endosomal sorting complexes required for transport (ESCRT) machinery [209,210]. This ESCRT machinery sequesters and sorts cytosolic ubiquitinated proteins into ILVs and is the most well-defined and widely understood system for biogenesis of normal and cancer exosomes. The ESCRT complex is comprised of a series of sub-complexes (i.e., ESCRT-0, ESCRT-I, ESCRT-II, and ESCRT-III) that are sequentially activated by binding of cytosolic ubiquitinated proteins (Figure 4), which are then internalized as early endosomes mature [211].

#### 4.1.3. MVB Trafficking and Exosome Release

The biogenesis of mature MVBs terminates with their progression through one of two pathways: (1) they fuse with lysosomes, resulting in the rapid and irreversible degradation of their contents, or (2) they fuse with the plasma membrane, resulting in the release of their ILV content into the extracellular environment, at which point they are referred to as exosomes [212]. The mechanisms underlying this intracellular switch, which determines the fate of MVBs, is complex and to our knowledge remains unclear. However, recent studies suggest that both paths are interconnected and that decisions affecting one result in alterations of the other [213]. For instance, two studies showed that inhibition of lysosome fusion with bafilomycin A1 results in the increased secretion of exosomes [214,215]. The release of exosomes by fusion of the MVB with the plasma membrane involves two sequential steps: (i) The targeted movement of MVBs to the plasma membrane, which is reliant on the structure of the microtubule cytoskeleton and the dynamic mechanisms of molecular motors, including dynein, kinesins, and actin-based myosin [216,217], and is regulated by several small Ras-like GTPases (including Rab27 A/B, Rab11, and Rab35), and soluble NSF attachment protein receptor (SNARE) (i.e., small, abundant tail-anchored proteins which are post-translationally inserted into the plasma membrane) proteins [218,219], which have been shown to differ between different cell types [220,221]. (ii) The fusion with the plasma membrane, wherein Rab11 and Rab35 facilitate MVB and plasma membrane fusion [222,223] through the assembly and functionalization of the “SNAREpin” complex. This complex is comprised of one vesicle-SNARE protein (located on MVBs) and three target membrane SNARE proteins (located on the plasma membrane) and is responsible for bringing the two membranes in close apposition resulting in the opening of a fusion pore and the merging of the MVB and plasma membrane [224] and their releasing their exosome cargo into the intercellular space.

### 4.2. Biological Functions of Exosomes

Exosomes are the most extensively characterized type of extracellular vesicles (typically 30–150 nm in size) [225], which, as described above, are produced via the endosomal pathway (Figure 1) [226]. The release of exosomes is an evolutionary process that has been conserved across all archaea, bacteria, and eukaryotic cells [227]. In humans, exosomes have been shown to regulate cellular functions of neighboring/target cells either by: (1) extracellular interactions of their membrane proteins and lipids with cell-surface receptors and membranes of recipient cells, respectively, to induce signaling cascades [228] or (2) cellular uptake and intracellular pre- or post-transcriptional reprogramming, via the uptake of transcription factors, microRNAs, and long non-coding RNAs, or via the import of foreign messenger RNAs, viral RNAs, and other RNA species [229,230]. Although initially thought to be responsible for the removal of unnecessary material (proteins, lipids, nucleic acids, etc.) [76], exosomes have been shown to play critical roles in a broad range of essential regulatory cellular functions, including the modulation of immunity [231] by regulation of immune cells, particularly by the inhibition of natural killer cells [232] or by acting as antiviral and antibacterial beacons [149,233]. Such activities have also been described with seminal fluid exosomes (originating from seminal glands, prostate, etc.), which protect (i.e., have antimicrobial activities) and provide energy (i.e., produce ATP) to sperm during fertilization [234]. Other functional studies of exosomes have identified their important roles in the maintenance of cellular stemness [235], coagulation of blood and repair of tissues [236], and cardiac and skeletal muscle repair [237,238]. Studies of the function of exosomes highlight the essential roles they play in maintaining the homeostasis of human tissues, which is perturbed during disease [239,240]. Unfortunately, the biological functions of exosomes are “hijacked” in human pathologies, including cardiovascular physiological and pathological disorders (e.g., cardiomyocyte hypertrophy, peripartum cardiomyopathy, and sepsis-induced cardiomyopathy) [241], where exosomes appear to contain erroneous cargoes. Furthermore, exosomes containing aggregation-prone proteins have been found in the cerebral spinal fluid and blood of Parkinson’s disease (PD), Alzheimer’s disease (AD), Creutzfeldt–Jakob disease (CJD), and amyotrophic lateral sclerosis (ALS) patients and may play roles in the propagation of these diseases [242,243,244,245]. Many studies have also highlighted the critical role that tumor exosomes play during the progression of cancer [246]. The best example on the functional diversion of exosomes has been described by the group of David Lyden, which showed that circulating tumor exosomes educate host cells within a secondary organ to prime that environment for establishment of the metastatic niche [247]. In their studies, they demonstrated that exosomes from mouse or human lung, liver, and brain tumor cells preferentially fuse with specific recipient cells (i.e., those at predicted target sites), namely lung epithelial and fibroblast cells, brain endothelial cells, and Kupffer cells in the liver [248].

### 4.3. Alteration of Exosome Biogenesis in Tumor Cells

Numerous studies have demonstrated that the presence of tumors is associated with a significant increase in the levels of circulating exosomes when compared to healthy individuals [249]. In aggressive brain cancer, glioma cells carrying the EGFRvIII overexpressing mutation exhibit increased secretion of exosomes harboring EGFRvIII [250], and when transferred to astrocytes can confer their oncogenic activity (i.e., ability of exosomes to convey their tumorigenic signals to recipient cells) [251].

**Figure 5:**
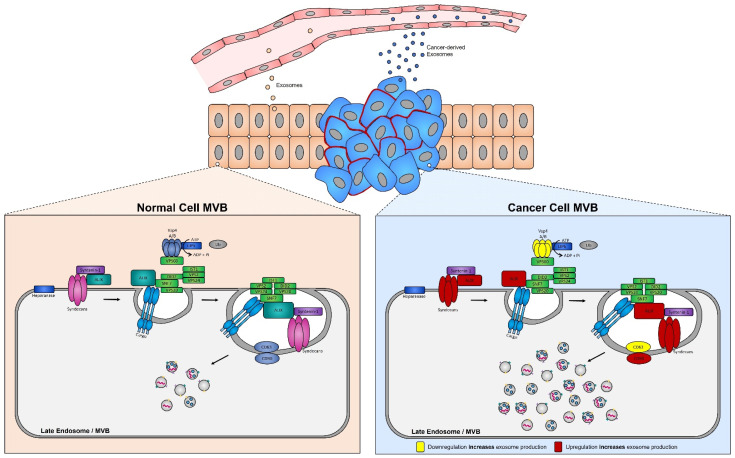
Syndecan–syntenin–ALIX couples to ESCRT-dependent MVB formation in healthy versus cancer cells. Syntenin-1 interacts directly with syndecans and ALIX, the interaction of ALIX with Snf7 of the ESCRT-III complex forms the syndecan–syntenin–ALIX pathway which is directly linked to exosome biogenesis. In cancer, several proteins in this pathway are altered, leading to enhanced exosome production. Alterations leading to the upregulation of either the syndecans, ALIX, and/or CD90 all result in enhanced exosome production, whereas any alterations leading to the downregulation of the ESCRT-III protein Vsp4 A/B and/or CD63 results in the increased production of exosomes seen in cancer cells.

ESCRT protein silencing: In order to elucidate the mechanisms underlying the increase in exosome biogenesis observed in cancer cells, Colombo et al. performed a set of iconic experiments to evaluate the role of the different ESCRT components in exosome biogenesis using Hela-CIITA-OVA ovarian cancer cells, which were modified to express MHC-II molecules and allowed for monitoring of exosome secretion [252]. Using this modified cell line, they carried out experiments to silence all twenty-three ESCRT proteins [252]. The silencing of ESCRT-0/I (STAM1, Hrs, and TSG101) resulted in decreased production of CD63, CD81, and MHC-II in exosomes, whereas silencing of ESCRT-III (Vps4B) or ALIX led to increased production and secretion of all exosomes [253]. Three other independent studies, where Hrs (an ESCRT-0 component) was silenced, also led to alterations in exosome secretion [254]. Specifically, the depletion of Hrs in non-cancerous dendritic cells led to a reduction in exosome secretion as measured by the detection of ubiquitinated proteins [254], whereas knockdown of Hrs in HEK293 kidney cancer cells and in head and neck squamous cell carcinoma led to increased exosome secretion and exosomal Wnt3a levels [255]. These studies demonstrated that alterations in ESCRT proteins directly influence exosome production, which may partially help explain their increased production in cancer cells (Figure 5).

Increased expression of ALIX in tumor cells: The most dramatic alteration in ESCRT-dependent exosome biogenesis for many cancer cell types, including colorectal carcinoma and pancreatic cancer [256,257], has been observed with the overexpression of ALIX, (i.e., induced by a ALIXΔPRR mutation) [258]. ALIX plays an essential role in exosome formation, through stabilization of ESCRT-III, facilitation of ILV formation, and cargo sorting [259]. Thus, upregulation of ALIX expression, as noted in many cancer cell types [260], partially explains the enhanced secretion of exosomes from tumor cells [256].

Increased expression of syntenin-1 in tumor cells: The expression of syntenin-1, which interacts directly with ALIX (via three LYPX_n_L motifs located within ALIX’s conserved cytoplasmic domains), is upregulated in several cancer types, including breast cancer, lung cancer, and melanoma [261,262,263]. Furthermore, quantitative proteomics identified syntenin-1 to be the most abundant protein present in these exosomes, suggesting that it may serve as a putative cancer biomarker [264]. Syntenin-1 has also been shown to interact with the N-terminus of syndecans (a small family of transmembrane proteoglycans) [265], thus linking them to exosome biogenesis through the syndecan–syntenin–ALIX pathway (i.e., syndecans recruit syntenin-1, which in turn binds to ALIX, and finally ALIX is recruited, stabilizing ESCRT-III and thus increasing exosome biogenesis) [266].

Increased expression of syndecans: Syndecans are a family of four core transmembrane proteins which typically carry two to five heparan sulfate chains [267]. Heparan sulfates are glycosaminoglycans that interact with a wide range of binding proteins, including cytokines, chemokines, growth factors, enzymes, and lipoproteins [268], and are regulated by the heparanase enzyme [269]. Increased expression of heparanase in tumor cells has been associated with increased cell aggressiveness (i.e., invasiveness and metastatic potential) [270]. Because heparanase activity is essential for the regulation of heparan sulfate chains of syndecans, and because syndecans are upregulated in several cancers [271], it is not surprising that heparanase is present in exosomes (ExoCarta, https://www.exoscarta.org/ (accessed on 29 May 2022). Indeed, an increased abundance of heparanase is seen in exosomes isolated from cancer patients compared to those from healthy individuals [272], suggesting enhanced exosomal release via the syndecan–syntenin–ALIX pathway [267,273].

## 5. Exosomes: A Source of Tumor Biomarkers

Valadi et al. were the first to determine that exosomes carry messenger RNAs (mRNAs) and microRNAs (miRNAs) [88], but since then many studies have demonstrated that exosomes contain a plethora of other RNA species (long-noncoding RNA (lncRNA), piwi RNAs, circular RNAs, etc.) [274]. Exosome whole-transcriptome studies have demonstrated that mRNA molecules are full length and well-protected from RNase activities within circulating exosomes [275]. Some studies have shown that exosomal mRNAs can be transcribed by recipient cells and are possibly used for reprogramming these cells [276]. However, the majority of exosome transcriptomic studies have been performed on microRNAs, because their function as post-transcriptional regulators allows them to control the recipient cells upon delivery [277]. Additional studies on the content of exosomes have also revealed the presence of fragmented genomic DNA, proteins, and lipids from both normal and cancer cells [278]. Fragmented genomic DNA, for example, which is more stable (i.e., protected from degradation) within circulating exosomes than in the circulation, can provide critical biomarkers for the detection of circulating tumor DNA mutations [279]. Castellanos-Rizaldos et al. recently showed that the detection of circulating genomic DNA carrying the EGFR^T790M^ mutation in patients with non-small cell lung cancer (NSCLC) was superior when isolated from circulating exosomes rather than circulating free DNA (cfDNA), particularly in patients with metastatic stage 0/1a lung cancer [126,280]. Overall, several free databases, including Vesiclepedia [281], EVpedia [282], ExoCarta [283] exoRBase [284], and EVmiRNA [285], have been made available for screening the transcriptomes, proteomes and lipidomes of exosomes from different cell types and tissues. For the purposes of this review, we will concentrate our evaluation of exosome biomarkers for known microRNAs, proteins, and lipids.

### 5.1. MicroRNA Biomarkers

MiRNAs represent a large class (~2600 identified in humans) of evolutionarily conserved small regulatory non-coding RNAs expressed from single genes, gene clusters, or intronic passengers, generally 19–25 nucleotides (nt) in length [277]. MiRNAs control gene expression by binding to imperfect complementary sites within the 3′ untranslated regions of their mRNA targets and orchestrate their degradation and/or post-transcriptional repression [286,287]. MiRNAs are involved in the control of all normal biological processes and their expression deregulation (i.e., increased or decreased expression) has been associated with the initiation and development of cancers [288,289,290,291]. Mature and functional cellular miRNAs can be detected in the blood and other biological fluids and can generally be found in two forms: either as cell/membrane-free molecules (i.e., free, bound to Argonaute (Argo) or to nucleophosmin proteins, complexed with high density lipoprotein (HDL)) or encapsulated within extracellular vesicles [274,291,292].

#### 5.1.1. Current Technologies for Quantification of Exosomal MicroRNAs

Since the cargo of exosomes is specifically tailored and to an extent reflects the state and condition of their cells of origin, circulating miRNAs have a potentially strong diagnostic and prognostic value as disease biomarkers [293]. Considering the low amount of material available from exosomes, which may be extracted from limited amounts of biofluids (saliva, sweat, urine, semen, exhaled breath condensates, etc.), current technologies and protocols have been optimized for the global quantification and detection of exosomal miRNAs [294].

##### High-Throughput Expression Analyses

Next generation sequencing offers a unique unbiased opportunity to globally evaluate the content of exosomes [295,296]. It is important to note that miRNA quantification of cell specific exosomes, either from culture media or biofluids, requires highly purified exosomes [149], because miRNAs are extremely robust [297] and any external contamination can significantly affect quantification. Most next generation miRNA sequencing protocols require relatively high quantities of starting material; for instance, the Illumina TruSeq small-RNA sequencing platform requires a minimum of 10–50 ng of purified small-RNA (Illumina TruSeq small-RNA protocol) or 1000 ng of total RNA [298]. As such, many studies have been focused on global exosomal miRNA purifications from blood or biofluids, which provide workable amounts of total RNA [296,299]. In these studies, exosomes have been rarely isolated from plasma volumes less than 1 mL, and typically these isolations are carried out by means of ultracentrifugation, which yields higher exosome quantities [112]. However, currently many researchers are driving the development towards the utilization of optimized protocols adapted for NGS analysis of low miRNA abundant exosome sub-populations [300]. To this end, our laboratory developed and optimized a cDNA library preparation protocol which allows for 3′ barcoded miRNAs from up to 18 individual samples to be pooled prior to 5′ adapter ligation, reverse transcription, and PCR amplification (~15 to 20 cycles decided via a pilot PCR reaction), which increases the carrier effect of the combined RNA for subsequent enzymatic reactions and purifications [301,302]. This protocol was purposely designed for the analysis of samples with minute quantities of total RNA, and we demonstrated its robustness for exosomal small-RNA analyses with total RNA input <1.5 ng [149]. Using the EV-CATCHER assay for highly pure selection of CD63 exosomes from the serum of patients infected with the SARS-CoV-2 virus and admitted to our health network, we were able to detect miRNA expression differences in miR-146a and miR-126-3p between COVID-19 patients hospitalized with mild symptoms that did not receive mechanical ventilation and COVID-19 patients hospitalized with severe respiratory symptoms that received mechanical ventilation, within levels estimated at ~25 zeptomoles (10 e-21 mole), which we successfully validated by qPCR [149]. Although the detection miRNAs from low-input total RNA is challenging, our experiments strongly indicate that the specificity and purity of the exosome preparation is critical for the successful detection of unique and rare disease miRNA biomarkers.

Of note, the Nanostring nCounter miRNA platform is an advancing technology for the sensitive, reproducible, and highly multiplexed analysis of up to 800 pre-selected miRNAs. The nCounter miRNA platform is based on a novel method of direct molecular barcoding and digital detection using color-coded probe-pairs [303]. This technology relies on the use of unique miRtags (i.e., oligonucleotide tags), which are ligated to the 3′ end of specific miRNAs. Sequence specificity during ligation is ensured through stepwise controlled annealing and ligation temperature in addition to the use of Tm-optimized bridging oligonucleotides complementary to a portion of the target miRNA and miRtag [304]. To date, a few studies have employed this technology for the quantification of miRNAs from plasma-derived ultracentrifuged exosomes [305]. This methodology is very attractive because it bypasses PCR amplification, which is required during the preparation of small-RNA cDNA libraries, but it may not offer the proper sensitivity yet for the analysis of molecules in the zeptamole concentration range.

#### Quantitative PCR (qPCR) Analyses

Currently there are numerous commercially available platforms and qPCR-based assays for the analysis of miRNAs (i.e., TaqMan miRNA assays (Thermo Fisher), TaqMan Array microRNA 384-well cards (Thermo Fisher, Tokyo, Japan), OpenArray (Thermo Fisher), SmartChip (Takara, Kusatsu City, Japan), miScript miRNA PCR arrays (Qiagen, Hilden, Germany), miRCURY™ LNA™ Universal RT microRNA PCR (Exiqon, Vedbæk, Denmark), and miRNA Oligo chip (3D Gene)). Quantitative PCR technology is still considered the gold standard for validation of findings made during high-throughput analyses; however, it has inherent biological and experimental limitations for exosomal miRNA studies, which include: (1) The discovery of tumor miRNA panels specific to a tumor exosome remains limited to known or selected miRNAs (which can be multiplexed for up to 700 miRNAs using TaqMan card arrays) rather than global unbiased NGS analyses. In this fashion it does not allow discovery of IsomiR (i.e., miRNA variants of the same miRNA, which can differ in sequence and/or length composition and will bind to different mRNA targets than the original miRNA sequence [306]) expression ratio differences for a specific miRNA in exosome sub-populations. The study of isomiRs is relevant in cancer, as their expression ratio has been shown to be affected by and can elicit changes in tumor cell migration and invasion [307]. (2) qPCR assays typically require large inputs of RNAs for global evaluation and may still require a pre-amplification step (i.e., typically ~14 cycles) before the 40-cycle amplification for fluorescent quantification, which leaves room for potential amplification bias or errors. Most importantly, even when using single qPCR assays, the sensitivity of detection may not be sufficient for miRNA expression validation of total RNA extracted from a selected subpopulation of circulating exosomes. In fact, we have previously demonstrated that the validation of differentially expressed exosome miRNAs, identified by NGS below 25 zeptomoles, using RT-qPCR is challenging as this quantification lies within the threshold of detection (Ct values of ~36–39) [149]. Studies that have been performed using total RNA extracted from globally circulating exosomes for NSCLC [308], prostate cancer [309], metastatic colorectal cancer [310], and gastric cancer [311] have all failed to provide robust, reproducible, and diagnostic cancer-specific miRNA signatures. The lack of technical standardization and the total RNA purification from globally purified exosomes from plasma (i.e., most of them likely released by blood cells) may have contributed to these limitations.

#### Droplet Digital PCR (ddPCR) Analysis

Droplet digital PCR (ddPCR) technology has thus become very popular because of its inherent capability for the ultra-sensitive detection of rare molecules [312]. This technology enables absolute quantification by partitioning the reaction into individual droplets (i.e., using water-in-oil and microfluidic technology), wherein molecules are randomly distributed, with each droplet/compartment containing zero, one, or many molecules. In essence this allows for amplification of each compartmentalized PCR mix/target molecule(s) to occur independently of each other [313]. Following amplification, the absorbance of each droplet is counted [314] and using Poisson statistics and the number of positive reactions (i.e., compartments which contain molecules and thus positive signal) the initial copy number and target density are calculated. ddPCR relies on the analysis of individual droplet amplitude and scatter for the absolute quantification of nucleic acids [315]. BioRad has become the leader in ddPCR technology and currently markets their award-winning QX200 AutoDG ddPCR instrument for either EvaGreen or probe-based digital PCR applications. The instrument’s specifications indicate that in each 100 µL volume, up to 20,000 droplets may be generated; generally data from 12,000–16,000 droplets are used for accurate quantification of target molecules (https://www.bio-rad.com/webroot/web/pdf/lsr/literature/Bulletin_6407.pdf (accessed on 25 May 2022)). Wang et al. recently demonstrated that the analysis of miRNA targets from exosomes (i.e., purified from urine), when compared with traditional TaqMan qPCR technology, was significantly more accurate and sensitive for the detection of low concentrations (i.e., <100 copies/µL) [316]. However, although Bio-Rad offers a large array of mRNA-based detection using their ddPCR instrument, they have not established a standardized protocol for the analysis of miRNAs, and the only available protocols are from published studies [317,318]. The protocol of Hindson et al. suggests that using ddPCR with TaqMan probes improved the day-to-day reproducibility by 7-fold, as seen by a 37–86% decrease in the coefficient of variation, compared to standard qPCR [315]. Considering that tumor exosome miRNA biomarkers may be within or below the zeptomole concentration in a given biofluid, ddPCR technology offers a unique opportunity for the development of ultra-low, -sensitive, and -specific clinical assays and the detection of rare tumor-specific circulating exosomal miRNAs. Standardization of both the tumor exosome isolation techniques (i.e., antigen-based capture, nanoparticle enumeration) and miRNA ddPCR assay preparation will be critical.

#### 5.1.2. Function of MicroRNA in Cancer Exosomes

Exosomal miRNAs from tumor exosomes have been shown to exert a multitude of biological effects during cancer progression which include: (1) the regulation of tumor growth, (2) The evasion from host immune responses, and (3) remodeling of the tumor microenvironment and metastasis.

##### Regulation of Tumor Growth

Cellular proliferation is a critical aspect to tumor progression, which commonly is a result of the deregulation of cell-cycle related proteins [316], and is a known hallmark of cancer [22]. Research studies have demonstrated that tumor-derived exosomal miRNAs can regulate cancer cell proliferation by targeting cell cycle-associated proteins and signaling proteins [317]. For example, in colorectal cancer, miR-200b is transferred between adjacent cancer cells, where it directly targets 3′-UTRs of p27 and Rho Family GTPase 3 (RND3), two proteins involved in cell cycle regulation [318,319], leading to their downregulation, which in turn induces proliferation in recipient cells [320]. Contrarily, miR-6869-5p, known to act as a tumor suppressor [321], is significantly downregulated in serum-derived exosomes originating from colorectal cancer cells [322]. The downregulation of exosomal miR-6869-5p delivery is associated with enhanced cell proliferation and increased production of the inflammatory cytokines interleukin-6 (IL-6) and tumor necrosis factor alpha (TNF-α) [323]. In metastatic breast cancer, the transfer of exosomal miR-1246 to non-malignant breast cells promotes proliferation, migration, and drug resistance through the direct downregulation of cyclin-G2 (CCNG2) [321]. Moreover, exosomal miRNAs profoundly regulate the apoptotic signaling pathway in cancer cells; for example, Jing et al. demonstrated that the delivery of exosomal miR-769-5p to gastric cancer cells directly targets caspase-9, a cysteine-aspartic protease that serves as the initiator of the intrinsic apoptosis pathway [324], leading to the inhibition of the downstream caspase-dependent apoptotic pathway, and promoting the degradation of the tumor suppressor protein p53 through the ubiquitin–proteosome pathway [325]. Furthermore, in breast cancer, the uptake of exosomal miR-128 leads to a reduction in the expression of the pro-apoptotic protein Bcl-2-associated X protein (Bax) in recipient breast cancer cells [326].

##### Evasion from Host Immune Responses

Exosomal miRNAs have been shown to function as critical mediators in the crosstalk between cancer cells and macrophages within the tumor microenvironment [327]. Macrophages can be divided into two classes: either M1-polarized or M2-polarized depending on their function [328]. M1 macrophages inhibit tumor growth whereas M2 macrophages have been demonstrated to provide immunosuppression around the tumor niche, while promoting tumor development and metastasis [329]. Exosomes secreted from the SKOV3 epithelial ovarian cancer cell line and containing miR-222-3p can induce macrophage polarization and differentiation to the M2 phenotype by suppression of the cytokine signaling 3 (SOCS3)/STAT3 pathway, which results in tumor growth and enhanced metastatic capability [330]. Similarly, exosomes from hypoxic ovarian cancer cells, present in the center of tumors, deliver miR-940 to macrophages and promote their polarization to the M2 phenotype, which results in tumor progression [331]. In a similar manner, hypoxic glioma-derived exosomes promote M2 macrophage polarization through the delivery of miR-1246, which targets TERF2 interacting protein (TER2IP) via the STAT3 and NF-κB pathways [332]. Dendritic cells are antigen presenting cells (APCs) linking innate and adaptive immunity which express a wide range of toll-like receptors (TLRs) and cytokines [333] that are critical for the activation of host immune responses to pathogens [334], including recognition of cancer cells [335]. In pancreatic cancer, the delivery of exosomes containing miR-203 can inhibit the expression of TLR4 and the production of cytokines, including TNF-α and interleukin-12 (IL-12) in dendritic cells [336], leading to dendritic cell dysfunction, which facilitates cancer cell evasion of the host’s immune response [337]. In addition to the effects that exosomal miRNAs have on macrophages and dendritic cells, they have also been shown to have profound effects on natural killer (NK) cells [338]. NK cells are a part of the innate immune system and can directly kill tumor cells [339] through the release of perforins and granzymes from their cytoplasm [340]. Briand et al. demonstrated that radiotherapy induced the release of exosomes containing increased levels of miR-378a-3p from a variety of cancer cell types, which resulted in the decreased release of granzyme-B from NK cells [341]. Natural killer group 2D receptor (NKG2D) is an activating receptor that is expressed on NK cells, which plays a pivotal role in tumor immunosurveillance [342]. Under hypoxic conditions, the IGR-Heu lung carcinoma and K562 myelogenous leukemia cell lines release exosomes containing increased levels of miR-23a, which when transferred to NK cells results in decreased expression of NKG2D, and thus reduced NK function [343].

##### Tumor Microenvironment Remodeling and Metastasis

The initiation of metastasis typically occurs when cancer cells undergo a process of epithelial-to-mesenchymal transition (EMT), wherein non-motile epithelial cells transform to a motile/invasive mesenchymal cell type [344]. Once these cells detach from primary tumors and travel through blood vessels, they establish the metastatic niche. Significant evidence suggests that tumor exosomes with their miRNA cargoes play a profound regulatory role in the metastatic process by controlling EMT, angiogenesis (i.e., formation of new blood vessels), niche cell education [345], and tumor cell invasion (Figure 1). Cai et al. demonstrated that exosomes isolated from glioblastoma patients carried increased levels of miR-148a compared to exosomes from healthy volunteers [346]. Exosomal delivery of miR-148a to T98G glioblastoma cells results in cell proliferation and metastasis by downregulation of the cell adhesion molecule 1 (CADM1) and activation of the STAT3 pathway. In triple negative breast cancer, exosomal miR-122 released from breast cancer cells has been shown to reduce the glycolytic pyruvate kinase PKM, and thus decreases glucose uptake in non-tumorigenic cells within the pre-metastatic niche, allowing for increased nutrient availability within the pre-metastatic niche and promoting survival of breast cancer cells that have metastasized [347]. In melanoma, tumor cells exchange miR-222 via exosomes to enhance tumor malignancy by the activation of the phosphoinositide 3-kinase (PI3K)/Akt pathway, via the downregulation of its target gene p27Kip1 [348]. Bone marrow-derived stem cells (BMSCs) present in the hypoxic tumor microenvironment are known to contribute to cancer progression [349]. In lung cancer, hypoxic BMSC-derived exosomes shuttle miR-193a-3p, miR-210-3p, and miR-5100 to lung epithelial cancer cells, which promotes their metastasis via the activation of STAT3 pathway-mediated EMT [350]. Moreover, Yang et al. demonstrated that hepatocarcinoma (HCC) cells with high metastatic potential are able to transfer exosomes containing miR-92a-3p to HCC cells with low metastatic potential, by promoting the EMT-mediated PEN/Akt pathway, as demonstrated by the increased expression of mesenchymal biomarkers (N-cadherin, β-catenin, Snail) and decreased expression of E-cadherin [275]. Altogether, these experiments demonstrate the powerful role that exosomes play in the intercellular communication of cancer cells for the self-propagation of cancer.

Altogether, these studies have demonstrated that cancer cells take advantage of exosomes to transfer tumorigenic miRNAs that can reprogram (i) adjacent tumor cells to promote tumor growth and (ii) a plethora of critical immune cells, to protect the tumor, and (iii) educate pre-metastatic niche cells for the formation of secondary tumor sites before metastasis. However, circulating cancer exosome studies conducted on the same human cancer types have not systematically identified the same deregulated miRNAs, globally suggesting that the miRNA packaging of exosomes may help differentiate healthy individuals from cancer patients (See Table 1). Along with the design of ultra-sensitive and specific cancer exosome purification assays, these studies highlight the potential clinical applications of exosome-based miRNA signatures for non-invasive diagnostic and prognostic evaluation of human cancers.

### 5.2. Protein Biomarkers

In addition to the many intact RNA species and fragmented genomic DNA available in exosomes, many proteins occupy the surface and interior compartments of these EVs. In the early days, the identification of protein components from EVs was performed by their separation on one-/two-dimensional gels by electrophoresis followed by tandem mass spectrometry [351,352,353,354]. Although these traditional approaches initially allowed for the identification of the mostly abundant proteins, it also allowed for the identification of important exosome protein biomarkers, such as tetraspanins (e.g., CD9, CD63, CD81, and CD151), 14-3-3 proteins, heat shock proteins (e.g., hsp70 and hsp90), and major histocompatibility complex (MHC) [351,352,353,354]. However, during the last two decades, great technical advances in proteomics have been made, which increased sensitivity and have allowed for the identification of thousands of proteins from exosomes and EV proteins obtained from various cell types and body fluids, including serum, urine, saliva, ascites, and breast milk, as shown in several databases (including EVpedia [282], ExoCarta [283], and Vesiclepedia [281]). The ever-growing list of proteins has largely deepened our understanding of EVs and facilitated the biomedical applications in multiple fields [355,356,357,358,359,360,361,362,363,364]. In this section, we will discuss the technological advances in EV proteomics and their applications in cancer liquid biopsy, particularly in the past several years.

#### 5.2.1. Protein Extraction from Exosomes and EVs Prior to Analysis

The isolation of exosomes or other EV species is critical to factor proteomic analyses, and it is easy to understand that sample quality directly affects the final proteomics results. Particularly, the excess of certain proteins may overwhelm the proteomic analyses of exosomes as exemplified with the evaluation of exosomes from cell culture media, which often contains a high abundance of albumin due to the addition of serum (i.e., bovine serum), or with exosomes contained in biofluids which carry large amounts of endogenous components (e.g., albumin or other abundant serum proteins from blood samples, uromodulin for urine samples). The presence of such confounding molecules often severely obscures the detection of EV proteins, especially low abundant ones. Therefore, specific procedures should be carefully designed to reduce these non-exosomal proteins or other contaminants, which interfere with proteomic analysis [365,366]. Of note, despite great interest in EVs and their associated proteins, there is currently no consensus on the preferred method(s) for EV isolation. Moreover, it appears that a single isolation method might not reveal the total and specific proteomic content of EVs [367,368]. It is anticipated that the combination of more than one method may be used to describe the proteome content of particular exosome populations, especially considering that exosomes circulating in biofluids represent a heterogeneous population of exosomes from different cellular origins [365,366,367]. Recently a comprehensive and quantitative proteomic analysis was performed on EVs isolated from human primary dendritic cells [369], and it was found that common exosome markers (such as MHC, flotillin, and HSP70) are all identified in all EVs independently of their sedimentation speed, whereas tumor susceptibility 101 (TSG101), syntenin-1, EHD4, Annexin XI, and ADAM10 appeared to be better protein markers for ectosomes than the traditional exosomal protein markers previously used. Undoubtedly, appropriate isolation methods should be adopted for a particular EV population of interest so that meaningful proteomics data can be achieved; the purification methods currently available for selection or purification of exosomes are described in a previous paragraph. As we suggested in this previous section, the combination of different purification methods may help achieve the adequate selection of exosomes from different types of biofluids [365,366,367,368].

#### 5.2.2. Exosome and EV Protein Processing Approaches

##### Traditional Methods

Unarguably, sample processing is a crucial step for successful proteomics studies.

With traditional proteomics workflows, proteins were often digested in gels or in solutions prior to analyses [351,352,353,354,365,366,367,368]. However, this approach suffers from several inherent drawbacks, such as tedious procedures (i.e., gel digestion and peptide purification/extraction) and lengthy processing time, which limits sample processing throughput and analytical reproducibility.

##### Filter-Aided Sample Preparation

As an alternative, filter-aided sample preparation (FASP), a simplified method for proteomics sample preparation, has been used for exosome and EV proteomics [370,371,372]. As a relatively new approach, suspension trapping (S-Trap)-based sample preparation has its own advantages [373]. In the S-Trap approach, samples are lysed and solubilized in 5% SDS to create fine particles so that they can be trapped in the filter column. Very strikingly, SDS and other contaminants can be easily removed into the flow-through (even more efficient than the FASP processing). Moreover, proteins retained on the column are readily digested into peptides, without an additional desalting process. All these advantages help further minimize sample loss and increase proteome coverage. Indeed, thorough comparison with the in-solution digestion processing and the FASP method suggests that the S-Trap approach provides the largest number of identifications and the highest reproducibility for EV proteomics [374]. Last but not least, the S-Trap approach allows analysis of substantially lower starting amounts of proteins (down to 50 ~ 200 ng), making it extremely appealing for characterization of minute amounts of EV samples.

#### 5.2.3. Mass Spectrometric Analysis of EV and Exosomal Proteins

##### Top-Down vs. Bottom-Up Proteomics

Due to the high throughput and its unbiased nature, modern mass spectrometry-based proteomics has allowed for the global identification and quantification of exosomes and other EV proteins [375,376,377,378,379]. Indeed, in the past few decades, quickly evolving proteomics techniques have played an instrumental role in analyzing EV proteins (especially exosomes and ectosomes). As one of the two strategies, top-down proteomics has been tentatively used for the characterization of EV proteins. For example, >200 proteoforms (including multiple proteoforms of the pro-inflammatory mediators S100 A8 and A9) were identified from exosomes shed by murine myeloid-derived suppressor cells [380]. In contrast, bottom-up proteomics has gained much popularity and is widely employed to characterize EV protein cargoes [375,376,377,378,379]. Instead of analyzing intact proteins, proteins are subjected to digestion and then analyzed by tandem mass spectrometry in the bottom-up proteomics strategy. Although almost all major proteomics workflows can be adapted for EV proteomes, challenges remain. Despite great endeavors having been made in the preparation of exosomes from cultured cells or from biofluids (as aforementioned), purification of large amounts of exosomes with high purity is still not routine, which is either a tedious task or inherently limited by the low biofluid volume output. To that end, there is a strong need to develop highly sensitive and robust sample processing procedures and quantification strategy methods so that deep proteomics for EVs can be achieved. Herein, we do not intend to summarize all proteomic techniques proposed; rather, we will briefly present key factors that determine the output of EV proteomics: the purity/quality of isolated EVs, EV protein sample processing procedures, and tandem mass spectrometry (MS/MS) techniques.

In addition to sample processing, mass spectrometry is another key aspect for EV proteomics. The tandem mass spectrometry-based EV proteomics is usually performed via the data-dependent acquisition (DDA) mode. By combining high-resolution mass spectrometers with isotopic labeling techniques, quantitative information can be obtained for exosome proteomics [375,376,377,378,379]. For example, stable isotopic labelling method using isobaric tags for relative and absolute quantitation (iTRAQ) has been used for urinary EV proteomics and the identification of potential prostate cancer biomarkers, with candidate proteins verified using multiple reaction monitoring (MRM) mass spectrometry (candidate proteins include fatty acid binding protein 5, granulin, AMBP, CHMP4A, and CHMP4C) [381]. Stable isotope labelling with amino acids in cell culture (SILAC) was applied for the proteomics of EVs derived from microsatellite unstable colorectal cancer, allowing for the identification of transforming growth factor beta receptor type 2 (TGFBR2)-regulated EV proteins (such as FN1, GLUL, CTGF, and CDK1) [382]. As a relatively newly emerged approach, label-free data-independent acquisition (DIA) such as SWATH (sequential window acquisition of all theoretical fragment ion) has also been used for the quantitative proteomics of EVs and exosomes [374]. In one study, DDA and SWATH-MS were combined to identify exosome biomarkers indicative of acute or persistent radiation-induced responses [383]. In comparison to total urine proteomics, urine exosome proteomics was demonstrated to be superior for the identification of radiation signatures. Moreover, 23 biomarkers were identified from urine exosomes and 24 biomarkers from serum exosomes post whole-body irradiation (WBI) [383]. Interestingly, proteomic signatures of urinary exosomes seemed to be different from those obtained in serum, with the former indicating injury of the liver, gastrointestinal, and genitourinary track whereas the latter indicated vascular injuries and acute inflammation in response to radiation. Recently, DIA-MS proteomics was used for the analysis of EVs extracted from breast cancer cells, with performance comparison between DDA-MS ad DIA-MS performed [374]. The analyses revealed that DIA-MS outperformed DDA-MS, with deeper EV proteomic coverage and better quantification accuracy achieved [374].

#### 5.2.4. Mass Spectrometric Analysis of EV and Exosomal Protein Modifications

Post-translational modifications (PTMs) add another layer to the regulation of protein functions [384,385]. In comparison to unmodified peptides, modified peptides are present in a low abundance and sub-stoichiometric ratio, making their characterization technically challenging. For such unique and low abundance peptides, enrichment (e.g., TiO2 for phosphopeptides and lectin chromatography for glycopeptides) is necessary prior to mass spectrometry analysis. So far, limited but very appealing studies have been performed for PTM proteomics of exosome samples. For examples, 19 phosphorylation sites corresponding to 14 phosphoproteins [386] and 126 N-glycopeptides belonging to 37 glycoproteins [387] were identified from human urine exosomes. In a recent study, by combining multi-lectin weak affinity chromatography and extensive fractionation (by using high pH reversed-phase chromatography), 378 glycoproteins with 604 glycosylation sites were identified from urine exosomes [388]. By analyzing exosomes isolated from the plasma of breast cancer patients and healthy subjects, a recent phosphoproteomics study identified 9225 and 1014 unique phosphopeptides, with 156 and 271 phosphorylation sites significantly regulated in the microvesicles and exosome fractions, respectively [389]. Among them, three potential phosphopeptide markers belonging to the RALGAPA2, PRKG1, and TJP2 proteins were found to be significantly different between breast cancer patients and healthy subjects. Recently, over 2300 phosphoproteins were identified in breast cancer EVs [390]. Among the identified proteins, many were found to be plasma membrane-based, further indicating that surface protein modifications on cancer exosomes are performed by imparting signaling functions to recipient cells (bypassing the need for the initiation of phosphorylations on membrane receptors). Collectively, although proteomics for PTMs has been largely under-studied in EVs, these very encouraging results suggest that PTMs of proteins should also be of great importance. Comprehensive characterization of protein PTMs in EVs will help understand the sorting mechanisms and identify novel PTM-specific functions of proteins secreted in EVs. They may also help with the discovery of novel biomarkers for translational applications as well as the identification of PTMs with therapeutic effects.

Taken together, proteomics has provided invaluable insights in all aspects of exosome research (i.e., EV biogenesis, secretion, and intercellular interactions). These analyses have revealed that exosomes are enriched with secreted proteins, plasma membrane proteins (e.g., adhesion proteins, receptors, tetraspanins, and transporters), vesicle trafficking-related proteins, cytoskeletal proteins (e.g., actins, myosins, and tubulins), and cytosolic proteins (such as molecular chaperones and metabolic enzymes), which play fundamental roles in their effects on recipient cells as is well studied with cancer metastasis [379,380,381,382,383,390,391]. It is also clear that EV proteomes are dependent not only on the parental cell type and conditions in which the EVs are secreted but are also based on the type of EVs. Very importantly for the diagnostic use of exosomes, the proteome content of each exosome population derived from different sources tends to retain unique signatures and markers of its origin (e.g., specific surface proteins), which may provide features for the early detection of cancer as a minimally invasive liquid biopsy approach.

### 5.3. Lipid Biomarkers

Lipids are diverse biomolecules that have a plethora of biological functions during developmental, physiological, and pathological processes [392,393,394]. Lipids provide structural integrity to cells and to their subcellular organelles, are an essential part of cellular organization, and allow intracellular trafficking [395,396,397]. Through direct and indirect signaling, lipids can regulate cellular proliferation [398], differentiation [399], and apoptosis [400] but also paly essential roles in energy homeostasis through mitochondrial fatty acid β-oxidation [401]. The dysregulation of lipids in cancer involves alterations of the membrane composition, up/down regulation of pleotropic signaling lipids, and changes in energy metabolism of the transformed cell [402]. These alterations have been linked to cancer cell growth [403], metastasis [404], immune evasion [405] and therapeutic resistance [394]. The study of exosome-derived lipids as cancer biomarkers is an emerging area of research. The lipidomic composition of cell-specific exosomes, their roles in exosome formation and biology, and the methods used for their analysis have been reviewed previously and thus will not be discussed in detail here [406,407,408,409,410,411]. A number of eloquent studies have also used lipidomic analysis to identify differences in cancer-derived exosome lipid composition compared to their parental cells [412,413,414,415]. While these studies are fundamental in understanding the composition of exosomes derived from diverse cancer cells, with an aim to elucidating their functional roles, their discussion is out of the scope of this review. This section will focus on the lipid profiles detected from cancer-derived exosomes that have demonstrated utility as diagnostic and prognostic biomarkers, which is a field in its infancy. All lipids presented in the forthcoming sections are annotated according to the nomenclature published by Fahy et al. [416,417].

#### 5.3.1. Methods for the Analysis of Lipids from Exosomes

Mass spectrometry-based technology is the most sensitive and widely used approach for the analysis of lipids from isolated exosomes [418,419,420]. In the forthcoming section we will provide a brief overview of the different direct and indirect MS methods used for the analysis of lipids from exosomes. Lipids can be analyzed directly from intact exosomes using matrix-assisted laser desorption ionization (MALDI) mass spectrometry [418]. Alternatively, they can be analyzed following one of the four routinely used solvent-based liquid–liquid extraction techniques using both direct and indirect MS methods [419].

##### Analysis of Intact Exosomes

This technique offers the most rapid and high-throughput analysis of exosomes because they are spotted directly onto the MALDI target without any prior sample preparation. Data acquisition can be carried out within minutes (per sample), providing results that have been shown to match those carried out by MALDI MS of lipid extracts [418]. MALDI is more tolerant to salt impurities when compared to other MS techniques, providing robust and sensitive detection of a wide range of lipid species [421]. A limitation of this technique is the inclusion of high abundant matrix ions that can complicate the resulting mass spectra and the suppression of low abundant lipids or lipids that do not ionize readily. This is particularly evident in positive ion mode, where phosphatidylcholines (PCs) predominate the spectra. There is, however, a number of sample preparation techniques that can be utilized for the targeted analysis of lipids, such as the lithium base-hydrolysis technique introduced recently by Tran et al. which mitigates these ion suppression effects, enabling the sensitive detection of a range of sphingolipids in positive ion mode [422]. Another limitation of both intact analysis and the direct analysis discussed in the following section is the inability of these techniques to separate isomeric and isobaric lipid ions, thereby reducing the number and identification of the lipids detected when compared to indirect methods of analysis. The ability to separate lipid isomers during direct MS analysis of lipids is being realized with the advancement of more sophisticated mass analyzers, including ion mobility and high-resolution platforms, that are enabling the separation of these previously unresolvable lipid ions when analyzed directly [423,424]. Ion mobility platforms employ an electrophoretic-based separation of ions in the gas phase based on their charge, size, and conformation [425]. In this way, ion mobility MS platforms enable the separation of complex lipid mixtures prior to mass analysis [426]. Aside from MALDI, another ion source that has utility for the intact analysis of isolated exosomes is desorption electrospray ionization (DESI). While DESI MS is yet to be utilized for the analysis of exosomes, this technique requires no sample preparation as the samples are placed directly in the ambient ion source whereby desorption and ionization of lipids from the sample are facilitated through a stream of charged solvent (electrospray) that is rapidly applied to the surface of the sample [427]. Thus, DESI MS offers promise as a high-throughput analytical technique of intact exosomes for biomarker investigations.

##### Direct MS Analysis of Lipid Extracts

The direct analysis of lipid extracts by electrospray ionization (ESI) coupled to tandem MS, without any prior chromatographic separation, was introduced as “shotgun lipidomics” by Han and Gross [428]. Using this technique, lipids are continuously infused into a mass spectrometer and analyzed using simultaneous MS and MS/MS scans, thereby providing structural information that enables their identification and, with the addition of internal standards, enables their quantification [414,429]. The separation of isomeric and isobaric lipids can again be seen as a limitation of this technique; however, with the advancements of the aforementioned instruments and the addition of differential ion mobility to the shotgun lipidomics workflow, the separation of isobars by direct infusion techniques is a now reality [430]. Direct analysis of lipids from exosomes is also possible using MALDI and DESI MS, as discussed for the analysis of lipids from intact exosomes.

##### Indirect MS Analysis of Lipid Extracts

Indirect analysis of lipids extracted from exosomes involves prior separation utilizing chromatography techniques coupled to MS analysis. Common chromatography techniques include gas chromatography, liquid chromatography, and thin layer chromatography [411,419,431]. The simplest and most cost-effective of these techniques is thin layer chromatography (TLC) coupled to MALDI MS [418]. With this technique, lipid extracts are separated based on their head group polarity using a stationary phase more commonly consisting of silica gel and an apolar solvent mobile phase [432]. The TLC plate is then stained to visualize the lipid bands, which can then either be excised for MALDI MS analysis following an additional lipid extraction protocol or analyzed directly using MALDI imaging [433]. Liquid chromatography-based MS (LC-MS) techniques are the most widely used of the indirect MS methods for lipid analysis [434,435]. As with TLC, the polarity of the mobile and stationary phases is selected based on the selectivity and sensitivity desired [436]. The separation of lipid classes based on their polar head group is more commonly carried out using hydrophilic interaction liquid chromatography (HILIC) coupled to MS [434]. For comprehensive subclass coverage based on lipid fatty acid composition, reverse-phase liquid chromatography (RPLC) is the method of choice [437]. The hydrophobic interactions of RPLC separates lipids based on their carbon chain lengths and levels of saturation, with longer chain polyunsaturated acyl-containing lipids eluting last [434]. The mobile phases used for the elution of lipids can greatly differ from lab to lab, but all are based around organic–aqueous compositions that modify hydrophilic/hydrophobic interactions.

#### 5.3.2. Lipidomic Profiling of Exosomes from Cancer Cells and Cancer Cell Lines

In a study of drug-sensitive vs. drug-resistant non-small lung cell cancer cells, Jung et al. used MALDI-ToF-MS to demonstrate that lipidomic profiles could stratify gefitinib-sensitive from gefitinib-resistant cells based on their exosome composition [438]. They detected 27 lipid signatures that were increased in resistant cells and 40 that were decreased when compared to their drug-sensitive counterpart. Lipids were identified as PCs and ether-linked PCs, lyso-PCs, sphingomyelins (SMs), phosphatidylglycerols (PGs), phosphatidylinositols (PIs), and lyso-PIs with varying fatty acid residues. In a comprehensive metabolic study of the pancreatic cell line PANC-1, the use of UPLC-ESI-Q-TOF-MS allowed identification of diacylglycerol (DAG) (20:2/18:1) as a biomarker of PANC-1 cells undergoing epithelial-to-mesenchymal transition (EMT) [420]. EMT is known to contribute to both metastatic disease and therapeutic resistance of pancreatic cancer cells [439,440]. These two studies demonstrate the utility of lipidomic exosome profiling for biomarker discovery, with potential to drive precision medicine by prediction of treatment response. LC-MS/MS analysis of EVs isolated from the prostate cell line, RWPE1, the prostate cancer cell line, NB26, and the metastatic/hormone resistant prostate cancer cell line, PC-3, showed similar lipid profiles when the mean abundance of lipid classes was compared [441]. Analysis of lipid subclasses and their fatty acyl residue composition from each sample, however, enabled the stratification of normal, cancer, and metastatic prostate cancer cells based on specific lipid profiles. In total, 187 lipid species were quantified as showing a differential abundance between these cell lines, highlighting the complexity of lipid analysis when considering full structural composition of each lipid class. DAG and triacylglycerol (TAG) species were decreased in tumor and metastatic cells when compared to their normal counterpart, with DG (16:0/22:6) detected with the highest fold change difference between normal vs. prostate cancer cells lines. Conversely, a number of PCs, SMs, phosphatidylethanolamine (PEs), ceramides (Cers), hexosylceramides (HexCers), phosphatidylserine (PS), PIs, cholesteryl esters (CEs), and ether-phospholipids (PLs), were enriched in both tumor and metastatic cell lines to degrees that were dependent on the structural combination of their subclass (head group) and fatty acid residues. A panel of biomarkers that could stratify prostate cancer and prostate cancer vs. metastatic disease was not proposed, possibly due to the number of lipids detected in this study and the processing method utilized to handle such complex data. Another extensive lipidomic study comparing the lipidomic profiles of exosomes from the ovarian cancer cell line, SKOV-3, to those detected in exosomes from normal ovarian surface epithelial cell line, HOSEPiC, identified 110 significantly altered lipids from over 1200 identified [442]. These again encompassed the phospholipid, sphingolipid, and sterol classes already mentioned in previous studies and these differences were dependent on the fatty acyl composition of the subclasses detected. The authors highlighted PG (34:1) and CE (18:2) as the lipids with the highest significance between the cell lines, along with PS (36:2) as a SKOV-3 cancer-specific EV lipid, and proposed the utility of these as potential biomarkers. Lipidomic analysis of EVs secreted from pancreatic ductal adenocarcinoma cells (PDACs) allowed detection of increased numbers of the pleotropic signaling lipid, ceramide-1-phosphate (C1P) [443]. While this was a functional study demonstrating that EVs containing C1P can promote pancreatic stem cell motility, the specificity of this sphingolipid subclass along with the report of C1P (d18:1/16:0) being the predominant species detected warrants further investigation for its use as a PDAC-specific EV biomarker.

#### 5.3.3. Lipidomic Profiling of Exosomes from Liquid Biopsies

Lipidomic analysis of exosomes isolated from patient plasma samples successfully classified early and late-stage non-small cell lung cancer (NSCLC) by using the 430 detectable lipid ions [444]. Using ultra-high resolution mass spectrometry without any prior chromatography separation, Fan et al. were able to discriminate the different stages of NSCLC vs. healthy individuals from 91 patient samples. The data were classified using two different multivariate statistical analysis methods, random forests (RF) and least absolute shrinkage and selection operator (LASSO) regression analysis. RF analysis identified a higher number of lipid signatures with discriminating capabilities, of which 15 were identified and validated as PC (18:1/18:2), PC (18:0/18:2), PC (16:0/22:6), PC (16:0/18:2), SM (d18:1/16:0), PC (18:0/20:3), PC (16:0/20:4), PC (16:0/22:5), CE (20:4), TAG (52:5), SM (d18:1/24:1), PC (18:0/18:1), PC (16:0/16:0), TAG (54:6), and LysoPC (16:0). Of the seven LASSO features detected with discriminatory capabilities, three were identified and validated and they overlapped with those detected during the RF analysis, which increased the power of these lipids as classifiers for diagnosing and stratifying NSCLC. The three overlapping species were PC (18:1/18:2), PC (18:0/20:3), and TAG (54:6). The authors of this study showed that despite the discriminatory area under the ROC (aucROC) curve values being higher for RF analysis, the specificity was higher when using LASSO and fewer lipid species. This study demonstrates the power of a relatively small number of lipid species as valid diagnostic and prognostic biomarkers for cancer detection. The analysis of lipid profiles from serum exosomes isolated from pancreatic cancer (PC) patients identified 20 lipid species that were able to discriminate PC from healthy controls [445]. By correlating the data on the dysregulated lipids with clinical data, this group was able to identify several lipid species that provided a prognostic value. LysoPC (22:0) was shown to correlate with tumor stage, diameter, lymphocyte count, and the diagnostic markers CA19-9 and CA242. PC (P-14:0/22:2) was also shown to correlate with tumor stage and lymphocyte count, but this lipid species also correlated with three diagnostic markers, CA19-9, CA242, and CEA. PE (16:0/18:1) was also associated with tumor stage and the CA19-9 and CA242 diagnostic markers. This lipid, however, was shown to be an independent prognostic factor that correlated with patients’ overall survival. In a study comparing the lipidomic profiles of exosomes isolated from urine samples taken from prostate cancer patients compared to healthy controls, Skotland et al. identified nine lipid species that were significantly different between the two groups [446]. HexCer (d18:1/16:0), LacCer (d18:1/16:0), and PC (16:0/18:1) were all found to increase in cancer exosomes relative to the control. Contrarily, PE (O-18:0/18:1), PE(P-36:4/O-36:5), PE (P-18:1/20:4), PS (16:0/18:1), PS (18:0/18:1), and PS (18:1/18:1) were found to decrease in cancer exosomes relative to the controls. Despite the statistical differences of these lipids between prostate cancer and healthy patient urinary exosomes, their diagnostic performance was poor when used alone, with sensitivity values ranging from 20 to 67%. However, when the authors processed their data taking into account lipid ratio comparisons, the ratios of LacCer (d18:1/16:0) to PS (18:1/18:1) and PS (18:0/18:2) significantly separated the prostate cancer from healthy controls with a sensitivity of 93% and a specificity of 100%. This study again provides evidence that a very small number of lipid species are capable of acting as diagnostic exosome-based biomarkers, but also that care needs to be taken during data analysis and interpretation to ensure rigor in the identification of lipid-based biomarker panels from liquid biopsies.

#### 5.3.4. Lipidomic-Specific Exosome Isolation for Use as Cancer Biomarkers

A number of studies have reported the utility of exosomes containing PS lipid species as diagnostic biomarkers. Although not technically lipidomic profiling analysis per se, PS-protein binding affinity assays have been used for the capture of cancer-based exosomes from cell culture and biological fluids [447,448,449,450,451]. In normal healthy cells, PS lipids are predominantly located on the inner leaflet of the lipid membrane, but in a diverse number of cancer cells, PS has been shown to externalize to the outer leaflet, and this has garnered interest for use as a novel biomarker and therapeutic target [452,453,454,455]. In a similar regard, tumor cell-derived exosomes also contain increased numbers of PS species that are predominantly located in the external membrane bilayer, where they are believed to play a role in the uptake of cancer-based exosomes by recipient cells [407,410,456]. In a proof-of-concept study, Lea et al. showed that PS-expressing exosomes detected from patient plasma could be used as a diagnostic biomarker for ovarian malignancies [457]. The group developed a stringent ELISA for selective binding and detection of PS-expressing exosomes that distinguished healthy individuals from patients with ovarian malignancies. The group also demonstrated the utility of their assay in detecting tumor recurrence from plasma-based liquid biopsies. This is a pivotal study as there are currently no standard diagnostic or prognostic biomarkers for ovarian cancers, and grading is often carried out following surgical resection and histological staining/analysis. The same group also utilized the PS ELISA-based assay system to quantify picogram amounts of PS-expressing exosomes from the blood of tumor-bearing mice [449]. In four preclinical model systems, the PS-expressing exosomes were detected weeks prior to the development of tumor masses, indicating that circulating PS-expressing exosomes could provide an excellent source of biomarkers for the early diagnosis of a number of tumors.

In summary, while the field of exosome-derived lipids as cancer biomarkers is in its infancy, the aforementioned studies demonstrate their promising utility as diagnostic and prognostic markers. The advancement of sophisticated MS instrumentation for the sensitive and high-throughput analysis of lipids will enable the rapid translation of data from the bench to the clinic. The biggest caveat is standardizing both MS workflows and the analysis of the complex lipid data from these studies. Standardized workflows, along with validated biomarker panels, are essential to enable the incorporation of exosome-derived lipids into clinical diagnostics.

## 6. Concluding Remarks

Advances in cancer diagnostic and therapeutic strategies have helped increase life expectancy. Indeed, molecularly guided early detection assays have improved personalized therapies and led the way toward better cancer care. Although advances in imaging technologies have greatly improved the detection of solid tumors, delays in cancer screening, generally due to the indolent nature of certain cancers (brain, pancreatic, lung, etc.), remain a major hurdle for improving prognosis. However, because tumors shed nucleic acids, proteins, lipids, and extracellular vesicles (i.e., exosomes) in their microenvironment and in the circulation, novel molecular strategies have emerged to enable non-invasive liquid biopsy screening. Highly sensitive and specific molecular assays for the detection of CTCs and ctDNA have provided the blueprint for the feasibility of liquid biopsies for non-invasive detection of solid tumors. Exosomes, whose biogenesis and cargoes are greatly altered in cancer cells, provide stably encapsulated and circulating molecular reservoirs of unique tumor biomarkers (i.e., proteins, RNAs, DNAs, and lipids), which are extensively studied for their potential to revolutionize the non-invasive detection of all human cancers. A diverse arsenal of methodologies and molecular assays has been designed to purify these exosomes, but to date many of these approaches (i.e., ultracentrifugation) still rely on global rather than cell-specific exosome selection. Incidentally, most circulating exosome studies aimed at non-invasively identifying cancer biomarkers have been performed with blood (i.e., serum or plasma) where exosome selectivity is necessary. Fortunately, during their biogenesis, exosomes and other extracellular vesicles inherit unique surface molecular fingerprints (i.e., proteins, lipids, etc.), specific to their cell of origin, which provide unique or uniquely combined targets for their immunoselection. A large body of circulating biomarker studies have been focused on evaluating exosome miRNA cargoes, because these non-coding small-RNA master regulators retain their function during transport and can still effectively reprogram/educate recipient cells after delivery. Although recent studies have shown that circulating cancer exosomes released by tumor cells contain unique miRNA ratios and species, most of them have not reached sufficient reproducibility for identification of actionable miRNA cancer biomarkers. It is because disease/tissue specific exosome sub-populations circulating in the blood represent a very small fraction of total circulating exosomes (i.e., blood cell exosomes), whose miRNA biomarker cargo detection is hindered by more abundant circulating contaminants. Using our EV-CATCHER assay, which was specifically tested and designed to improve the selectivity and purity of immunopurified extracellular vesicles prior to their miRNA analysis, we demonstrated that isolation is indeed the most critical step for identification of rare miRNA biomarkers (i.e., circulating contaminants quench low specific signals), especially when using blood as the source of exosomes. Given the stability of exosomes as well as the limited release of contaminants, of the evaluation tissue-specific biofluids, such as urine for bladder cancer, saliva for oral cancer, exhaled breath for lung cancer, and tears for ocular melanoma may allow for more concentrated but most importantly non-invasive access to cancer exosome biomarkers.

Currently, the lack of standardization for exosome isolation and purification of their analytes remains the biggest hurdle for reproducible cancer exosome biomarker identification. While certain guidelines have been established for the characterization of exosomes and other extracellular vesicles [458], the fact that multiple isolation techniques are available to researchers along with the lack of established standardized techniques for the highly sensitive quantification of their low abundance cargo (i.e., nucleic acids, proteins, and lipids) reveals that to date no FDA-approved exosomal biomarker signature is clinically available for the detection and monitoring of any cancer type.

## Figures and Tables

**Table 1:** Published miRNA exosome cancer biomarkers identified in human biofluids. Lists of exosomal miRNAs circulating in biofluids of patients diagnosed with colorectal cancer, ovarian cancer, Glioblastoma, liver cancer, pancreatic cancer, lung cancer, extranodal natural killer/T-cell lymphoma, and prostate cancers.

Cancer Type	Differentially Expressed Between Healthy and Cancer		Reference
	miRNA Biomarkers		
Colorectal Cancer	↑	miR-224-5p, miR-548d-5p, miR-200a-3p, miR-320d, miR-200b-3p, miR-1246	Tang et al., 2019 [310]
	↓	novel_246, novel_301, miR-27a-5p	
		miR-135a-5p, miR-204-5p	Sun et al., 2021 [104]
	↓	miR-6869-5p	Yan et al., 2018 [321]
	↑	miR-486-5p, miR-3180-5p	Yan et al., 2017 [322]
	↓	miR-638, miR-5787, miR-8075, miR-6869-5p, miR-548c-5p	
Ovarian Cancer	↑	miR-21, miR-141, miR-200a, miR-200b, miR-200c, miR-203, miR-205, miR-214, miR-215	Taylor and Taylor, 2008 [89]
	↑	miR-940	Chen et al., 2017 [331]
	↑	miR-222-3p	Ying et al., 2016 [330]
Glioblastoma	↑	let-7a, miR-15b, miR-16, miR-19b, miR-21, miR-26a, miR-27a, miR-92, miR-93, miR-320, miR-20	Skog et al., 2008 [91]
	↑	miR-148a	Cai et al., 2018 [346]
Liver Cancer	↑	miR-17, miR-18a, miR-19a, miR-19b, miR-20a, miR-92a-3p	Yang et al., 2020 [276]
	↑	miR-193a-3p, miR-210-3p, miR-5100	Zhang et al., 2019 [350]
Pancreatic Cancer	↑	miR-21, miR-210	Wu et al., 2020 [294]
	↑	miR-193a-3p, miR-210-3p, miR-5100	Zhang et al., 2019 [350]
Lung Cancer	↑	miR-132-3p, miR-181b-5p, miR-27a-3p, miR-27b-3p, miR-320a, miR-361-5p, let-7b-5p, miR-24-3p, miR-3184-5p, miR-486-5p, miR-486-3p, miR-320b	Jin et al., 2017 [295]
	↓	let-7a-5p, let-7d-5p, let-7f-5p, miR-26b-5p, miR-30a-3p, miR-30e-3p, miR-744-5p, miR-744-5p, let-7e-5p, miR-191-5p, miR-191-5p, miR-206, miR-21-5p, miR-23a-5p, miR-23b-5p, miR-10b-5p, miR-15b-5p	
		miR-30b, miR-30c, miR-103, miR-122, miR-195, miR-203, miR-221, miR-222	Giallombardo et al., 2016 [308]
	↑	miR-193a-3p, miR-210-3p, miR-5100	Zhang et al., 2019 [350]
Extranodal Natural Killer/T-Cell Lymphoma	↑	miR-320e, miR-4454, miR-4516, miR-630, miR-122-5p, miR-574-5p, miR-22-3p, miR-486-3p, miR-1915-5p, miR-1972, miR-1285-5p, miR-222-3p, miR-1305, miR-891b, miR-4455, miR-21-5p, miR-1258, let-7b-5p, miR-25-3p, miR-1268a	Ryu et al., 2020 [305]
	↓	miR-564, miR-196a-5p, miR-520c-3p, let-7d-5p, let-7i-5p, miR-212-3p, miR-29a-3p, miR-608, miR-503-5p, miR-587, miR-548g-3p, miR-765, miR-34c-3p, miR-770-5p, miR-301a-5p, miR-526a, miR-340-5p, miR-325, miR-199a-3p+miR-199b-3p, miR-423-3p	
Prostate Cancer	↓	miR-196a-5p, miR-34a-5p, miR-501-3p, miR-92a-1-5p	Rodríguez et al., 2017 [309]

↑ = miRNA expression observed in circulating exosomes isolated from cancer patients as compared to circulating exosomal miRNAs isolated from healthy individuals and ↓ = decreased miRNA expression observed in circulating exosomes isolated from cancer patients as compared to circulating exosomal miRNAs isolated from healthy individuals.
